# Motor ability, physical self‐concept and health‐related quality of life in pediatric cancer survivors

**DOI:** 10.1002/cam4.3750

**Published:** 2021-02-01

**Authors:** Valentin Benzing, Valerie Siegwart, Janine Spitzhüttl, Jürg Schmid, Michael Grotzer, Claudia M. Roebers, Maja Steinlin, Kurt Leibundgut, Regula Everts, Mirko Schmidt

**Affiliations:** ^1^ Institute of Sport Science University of Bern Bern Switzerland; ^2^ Division of Pediatric Hematology and Oncology University Children’s Hospital Bern, Inselspital Bern University Hospital University of Bern Bern Switzerland; ^3^ Division of Neuropaediatrics Development and Rehabilitation University Children’s Hospital Bern, Inselspital Bern University Hospital University of Bern Bern Switzerland; ^4^ Institute of Psychology University of Bern Bern Switzerland; ^5^ Division of Pediatric Oncology University Children’s Hospital Zurich Zurich Switzerland

**Keywords:** childhood cancer, motor functioning, motor performance, pediatric oncology, perceived motor competence, physical fitness, well‐being

## Abstract

**Background:**

Cancer survivorship is frequently associated with severe late effects. However, research into pediatric cancer survivors on late effects in motor ability, physical self‐concept and their relationship to quality of life is limited.

**Methods:**

Using multiple regression analyses, 78 pediatric cancer survivors and 56 typically developing children were compared in motor ability, physical self‐concept and health‐related quality of life. In addition, mediational multi‐group analyses between motor ability (independent variable), physical self‐concept (mediator) and quality of life (dependent variable) were calculated.

**Results:**

Pediatric cancer survivors had a lower motor ability (*g*_Hedges_ = 0.863), a lower physical self‐concept with regard to several scales of the PSDQ‐S (*g*_Hedges_ = 0.318–0.764) and a higher relative risk for a below average quality of life than controls (*RR* = 1.44). Children with a history of cancer involving the central nervous system showed poorer motor ability compared to those without central nervous system

involvement (*g*_Hedges_ = 0.591). Furthermore, the physical self‐concept significantly mediated the relationship between motor ability and quality of life in pediatric cancer survivors but not in typically developing children.

**Conclusions:**

Results show the importance of monitoring and supporting the development of motor ability in the aftercare of pediatric cancer survivors. Physical activity interventions may be advisable to prevent physical activity‐related late effects and potentially improve related psychosocial variables such as quality of life.

## Graphical abstract

This study shows that pediatric cancer survivors suffer from a lower motor ability, a lower physical self‐concept and a higher probability for a below average quality of life than controls. Both the motor ability and the physical self‐concept are related to quality of life, indicating that motor ability needs to be monitored in the aftercare of survivors of pediatric cancer.

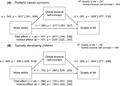

## 1 INTRODUCTION

Due to cancer and its treatment, pediatric cancer survivors (PCS) are a vulnerable group at high risk for late effects.^1^ Late effects cover a broad range of physical and psychosocial domains such as skeletal maturation, physical activity levels and self‐esteem.^1^, ^2^ Physical and psychosocial late effects, which were found to be interrelated in PCS,^2^ may therefore contribute to a high burden of disease and a lower quality of life (QoL).^3^, ^4^, ^5^

In typically developing (TD) children, there is strong evidence that physical activity is related to mental health and QoL.^6^, ^7^, ^8^, ^9^ Also in child and adolescent cancer survivors, exercise is increasingly considered important as part of routine cancer care.^10^ However, it is not only the physical activity level itself, but also the associated motor competence which seems critical to promote health trajectories.^11^, ^12^, ^13^

As indicated in Stodden´s (2008) comprehensive conceptual model, the development of motor competence is crucial in childhood because it enables children and adolescents to participate in different types of physical activities.^13^ This assumption is supported by empirical evidence, linking motor competence (as a mediator) to actual and future physical activity levels, physical fitness, and weight status.^12^ Furthermore, motor competence is considered key for successful physical, social and cognitive development.^14^, ^15^, ^16^, ^17^

However, research has shown that PCS are less physically active and have lower motor abilities than their peers.^18^, ^19^, ^20^ For example, there is evidence for poorer aerobic fitness, strength, balance, and coordination in PCS.^21^, ^22^, ^23^ Such deficits are not only reflected in objective assessments, but also in subjective reports of performance limitations.^24^, ^25^

The physical self‐concept, defined as the subjective self‐evaluation of one's physical attributes in areas of physical ability and appearance, is a hierarchically organized and multidimensional construct.^26^ It has been found to be associated with actual motor competence and to mediate the relationship between actual motor performance and physical activity.^27^, ^28^, ^29^, ^30^, ^31^, ^32^ It has been increasingly investigated because it is related to physical activity participation and considered central to health and well‐being.^33^, ^34^, ^35^ It has, however, not been investigated systematically in PCS to date.^36^

The first aim of this study was to investigate whether motor ability, physical self‐concept and QoL are lower in PCS compared to TD children. Against the background of poorer aerobic fitness, strength and coordination ^21^, ^22^, ^23^ in PCS, we expected to detect poorer motor ability in PCS compared to TD children. Considering the meta‐analytical results showing a lower self‐concept in children and adolescents with chronic health conditions,^37^ and the results of a recent systematic review indicating a reduced QoL in PCS,^4^ we expected lower physical self‐concept and QoL ratings in PCS compared to TD children. Related to the burden of disease, which is highest in survivors of malignancies involving the central nervous system (CNS),^3^ we expected PCS after cancer not involving the CNS (non‐CNS) to have better scores in physical self‐concept and QoL than PCS with CNS involvement. The second aim of this study was to investigate the relationship between motor ability, physical self‐concept and QoL in PCS compared to TD children. We hypothesized that the global physical self‐concept mediates the relationship between motor ability and QoL in PCS and TD children. This hypothesis is grounded on piecemeal evidence with regard to: (a) the conceptual model by Stodden et al.^13^; (b) the relationship between the physical self‐concept and actual motor performance in TD children^27^, ^28^, ^29^; and (c) the importance of the physical self‐concept for health and well‐being.^32^, ^33^

## 2 METHODS

### 2.1 Design and procedures

This study includes data of the Brainfit study, a randomized controlled trial (RCT) conducted in the cantons of Bern and Zurich, Switzerland.^38^, ^39^ For this study, background variables, motor ability, physical self‐concept, and health‐related QoL ratings of the first measurement point of the RCT were used. Children who did not wish to participate in the longitudinal RCT (e.g., because of the time‐consuming study design) but agreed to a single measurement point were additionally included in this study. Assessments were conducted in Bern and Zurich. Investigators conducting the assessments were blinded.

### 2.2 Participants

Participants were recruited at two specialized pediatric university hospitals in Bern and Zurich, Switzerland. Considering the inclusion and exclusion criteria (described below), a list of eligible survivors was provided by the Swiss Childhood Cancer Registry (SCCR). Out of 262 successfully contacted PCS, 20 did not meet the inclusion criteria at the time of recruitment (e.g., relapse), 161 declined to participate (e.g., the travel distance to the study location was too far, participation required too much effort, current health status, and a lack of interest) and 81 agreed to participate in this study. PCS who declined and who agreed to participate did not differ with regard to demographic and clinical variables (see Table S1). For study inclusion, participants had to be aged between 7 and 16 years and diagnosed with cancer within the past 10 years, including cancer both with or without CNS involvement (i.e., brain tumor, spinal cord tumor or leukemia). The cancer treatment (surgery, radiation, and/or chemotherapy) had to be terminated at least 12 months prior to participation in order to assess long‐term sequelae of childhood cancer. In addition, if the cancer did not involve the CNS, treatment had to include either radiation or chemotherapy in addition to surgical removal of the tumor. Survivors with secondary, benign, and malignant tumors were included. Exclusion criteria were: (a) any unstable health condition, (b) substance abuse, and (c) inability to follow study procedures. Furthermore, participants and their parents were informed that noncompliance during the study would lead to study exclusion. The data of three PCS were excluded from analyses (relapse: *n* = 1, noncompliance: *n* = 1, language problems: *n* = 1). Thus, the final sample size included 78 PCS. In addition, 56 TD children and adolescents participated in this study. These children were recruited via siblings of the patients (*n* = 2) and through public notice boards. Inclusion criteria were: (a) age between 7 and 16 years, (b) no history of neurological disease or cancer, (c) no mental or chronic disorders, (d) no developmental disorders (e.g., autism), (e) unimpaired hearing and vision. Exclusion criteria as for PCS were applied to TD children.

### 2.3 Measures

The following *background variables* were assessed: Age and sex were recorded from questionnaires. Height and weight were measured with a tape rule and a scale. Information about socioeconomic status was gathered using an adapted version of the family affluence scale^40^ (as reported by the parents). The family affluence scale consists of four questions regarding the family wealth (e.g., whether their child has its own bedroom, the number of family‐owned cars etc.). The response format varies from item to item, and points are given for example for the number of family‐owned cars. The sum of the four items ranges between 0 and 9 and constitutes the prosperity index. An acceptable reliability and validity has been demonstrated.^40^ Information about physical activity behavior was gathered using an adapted version of the Physical activity, Exercise, and Sport Questionnaire.^41^ Parents had to indicate the frequency and duration of up to three types of exercise that their child regularly engages in, resulting in an average number of minutes per week. Acceptable psychometric properties have been demonstrated.^41^ Nonverbal IQ was assessed using age‐based standard scores (*M* = 100, *SD* = 15) of the test of nonverbal intelligence (TONI‐4), fourth edition.^42^ Age at diagnosis, cancer type, treatment duration, and type of treatment were derived from the SCCR and in case of missing information verified using clinical records.

Motor ability was assessed using five items of the German Motor Test including coordination (balancing backwards, jumping sideways) and strength (sit‐ups, push‐ups, long‐jump)^43^ and a cycle ergometer test. In the five items of the German Motor Test participants either have a time limit (jumping sideways, sit‐ups, push‐ups) or two trials (balancing backwards, long‐jump), in which their performance is measured. The derived performance raw scores were subsequently transformed to a standardized (age and gender specific) *Z*‐score (*M* = 100, *SD* = 10) using the formula *Z* = 100 + 10 *×* $\frac{x\;‐\;\mu }{\sigma }$. *Z*‐scores range from 70 to 130 and the total score was calculated from the mean *Z*‐scores of the five items applied. The total score, as well as the coordination and strength score were used for analyses. The population means and standard deviations of the German Motor Test are based on a reference population which was representative for Germany, including 4529 German children and adolescents between the ages of 4–17 years.^43^ According to the test manual, *Z*‐scores of 97.5 and below are considered as below average; scores between 97.5 and 102.5 are considered as average; scores above 102.5 are considered as above average. For this test, an acceptable validity (content validity: expert survey; construct validity: exploratory and confirmatory factor analyses, with acceptable model fit for the latter; criterion validity: correlation with teacher rating *r* = .69) and test‐retest reliability with a test interval of eight days (*r* = .82) has been demonstrated.^43^ For aerobic fitness a cycle ergometer test was conducted using the Godfrey protocol^44^ and the aeroman**^®^** professional (Aceos, Fürth, Germany). Because of technical problems with the device, 24.3% of the data was missing. Therefore, the maximal workload (power in watts), which was not affected by the technical problems, was used for analyses. In the complete data, a positive relationship between maximal oxygen uptake and maximal workload was found using Spearman's rank‐order correlation (*r* = .87, *p* < .001). The pattern of results did not change when relative VO2max was used in the reduced sample.

Physical self‐concept was measured using the German version of the short form of the Physical Self‐Description Questionnaire (PSDQ‐S).^26^, ^45^ This 40‐item questionnaire consists of nine specific component scales (health, coordination, activity, body fat, sports competence, appearance, strength, flexibility, and endurance), as well as of a global physical self‐concept and a global self‐esteem scale, which were used for the analyses. A sample item is: “Physically, I am happy with myself”. Answers have to be given on a 6‐point Likert scale ranging from 1 (strongly disagree) to 6 (strongly agree). Each scale includes the mean of 3–5 items (depending on the scale). A higher score represents a better physical self‐concept. Factorial invariance with an acceptable test‐retest reliability with a test interval of one year (median of the 11 scales: ﻿*r* = .77﻿), good convergent validity in relation to both time (mean = 0.80) and two other physical self‐concept instruments (mean *r*s* *= 0.81), and good discriminant validity (mean *r*s* *= 0.37), has previously been demonstrated.^26^

Health‐related QoL was measured using the parent version of the KIDSCREEN‐10,^46^, ^47^ which is based on the construct of QoL including physical, emotional, mental, social, and behavioral components of well‐being and functioning. The KIDSCREEN‐10^46^, ^47^ is a unidimensional questionnaire consisting of 10 items. Answers are given on a 5‐point Likert scale ranging from not at all to very/extremely (item 1 and 9) and never to always (other items). For analyses, items were coded so that higher values indicate better QoL and they were subsequently summed up. Gender‐ and age‐specific Rasch person parameters (which were transformed into values with a mean of 50 and a standard deviation of approximately 10) were assigned to each sum score. These scores were transformed into a *T*‐score considering the Swiss reference population and subsequently used for analyses. The Swiss population mean and standard deviation of the KIDSCREEN‐10 are based on a reference population which was representative for Switzerland, including 1701 Swiss children and adolescents between the ages of 8 and 18 years and their parents.^48^ An acceptable test‐retest reliability (test interval: 4 weeks; *r* = 0.70), internal consistency (Cronbach's alpha = 0.82), has previously been demonstrated.^46^ For convergent validity the KIDSCREEN‐10 was correlated to other validated questionnaires measuring similar constructs finding strong correlations (*r*s > 0.61) with the Youth Quality of Life Instrument‐Surveillance Version and the Child Health and Illness Profile (for further details and the manual see: https://www.kidscreen.org). High scores represent a good QoL. According to the manual scores of ≤40 are considered as potentially clinically significant values.

### 2.4 Statistical analyses

Because of a total amount of missing data of 14.3% in the main outcome variables of this study, between‐group comparisons were analyzed using a multiply imputed dataset. Data were imputed in SPSS (five imputations), applying fully conditional specification (predictive mean matching).^49^ Fully conditional specification was based on all available variables of the dataset. Reasons for missing data were (a) unreturned questionnaires; (b) motor ability assessment could not be terminated because of time constraints; (c) one person could not participate in the motor ability assessment because of a muscle injury. For analyses using multiple imputed data, pooled parameters are reported.

To compare background variables between PCS and TD children and between PCS after non‐CNS cancer and PCS after CNS cancer, independent *t*‐tests (two‐tailed) and *χ* ^2^‐tests were calculated.

For the analyses of dependent variables, multiple regression analyses using Helmert contrast coding were performed.^50^ This contrast is an orthogonal analysis, allowing to investigate the differences between PCS and TD children and the differences between PCS of non‐CNS and CNS cancer. Considering recommendations to present covariate‐free results and the controversial discussion about the “misunderstanding” of analysis of covariance,^51^, ^52^ first, covariate‐free analyses (model 1) were conducted; second, potential confounders (age, sex, socioeconomic status) were included in the model (model 2). In addition, the relative risk (*RR*)^53^ for a below average motor performance (≤97.5) and a potentially clinically significant score in QoL (≤40) were calculated by dividing the probability of the event in the exposed group by the probability of the event in the not exposed group ($RR\;=\frac{a\;/\;\left(a+b\right)}{c\;/\;\left(c+d\right)}$).

**FIGURE 1:**
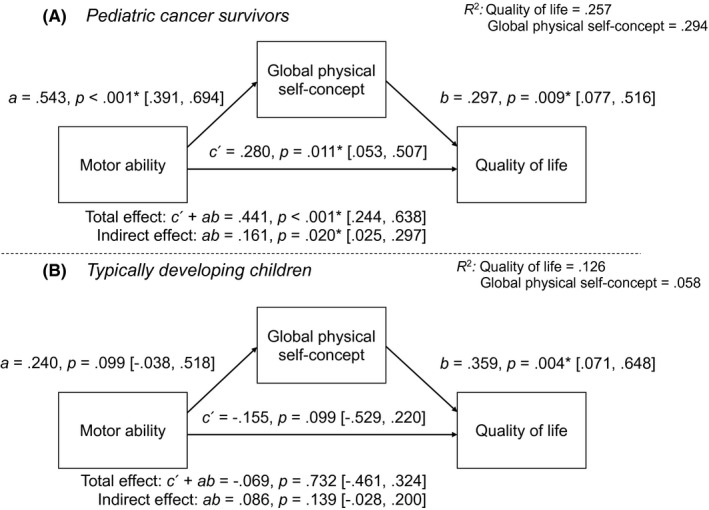
Mediation analyses between motor ability (German Motor Test), the physical self‐concept (PSDQ‐S global physical) and quality of life (KIDSCREEN‐10) for pediatric cancer survivors (1A) and typically developing children (1B). Note. R^2^ represents the proportion of the explained variance by the model; a, b, c’ refer to the respective paths of the mediation model; for each path standardized parameter estimates, significances and standardized confidence intervals (in square brackets) are indicated; path c’ represents the direct effect

To test whether a potential relationship between actual motor ability (total score) and QoL was mediated by physical self‐concept, mediation analyses were performed in R^54^ using the lavaan package.^55^ Motor ability (total score) was set up as independent variable, QoL as dependent variable and the global physical self‐concept as mediator. Maximum‐likelihood estimation with robust (Huber‐White) standard errors was used and missing values were estimated using full information maximum‐likelihood estimation. First covariate‐free multi‐group analyses (PCS and TD) were conducted (see Figure 1). Second, potential confounders (age, sex, socioeconomic status) were added to the models (Figure S1). Third, since descriptively the pattern of results did not change between the covariate‐free and the model including potential confounders, multi‐group analyses were performed to compare estimates between the two groups using the covariate‐free model: One model (1) in which the regressions paths were freely estimated across the two groups, three models (2.1–2.3) in which a single regression path was set equal (a, b, or c’), three models (3.1–3.3) in which two regression estimates were constrained to be equal (a and b, a and c’ or b and c’), and one model (4) in which all three regression paths were constrained to equality. Model comparison was made using both model fit indices (CFI, RMSEA, AIC, BIC) and the Satorra‐Bentler scaled chi‐square difference tests.^56^ All path coefficients of the mediation analyses are presented as standardized estimates.

Hedges *g*,^57^ which is interpreted similar as Cohen's *d*, taking into account the pooled and weighted standard deviation, was reported as an estimation of effect size (small effect size = 0.2, medium effect size = 0.5, large effect size = 0.8). For multiple regression model summary, in addition Cohen's *f* ^2^ is reported (small effect size = 0.02, medium effect size = 0.15, large effect size = 0.35).^58^ Level of significance was set at *α* = 0.05 for all analyses.

## 3 RESULTS

### 3.1 Background variables

Socio‐demographic background variables (see Table 1) did not differ between PCS and TD children. On average, survivors of CNS cancer were found to be older at diagnosis and had a shorter duration of treatment than those after non‐CNS cancer (see Table 2). These findings are consistent with incidence rates known from the literature (a proportionally higher incidence of CNS tumors in age 5–9 compared to age 0–4)^59^ and with the high percentage of treatment including only surgery and therefore a shorter treatment duration observed in our data. Therefore, both variables are inevitably linked to the cancer type and were not included as covariates for the comparisons of survivors of non‐CNS and CNS cancer.

**TABLE 1:** Characteristics of study participants

	Controls (*n* = 56)	PCS (*n* = 78)	
	Mean (*SD*)	Mean (*SD*)	*p*
Socio‐demographic background variables			
Age [years]	11.49 (2.75)	11.23 (2.49)	0.565
Sex [female/male]	27/29	32/46	0.408
Height [cm]	147.92 (17.89)	145.11 (14.62)	0.338
Weight [kg]	41.92 (17.69)	40.74 (14.17)	0.686
Socioeconomic status [0–9]	6.84 (1.49)	6.6 (1.43)	0.446
Physical activity behavior [minutes/month]	702.95 (822.13)	650.54 (643.71)	0.713
Nonverbal IQ^a^	107.36 (12.22)	105.99 (11.69)	0.291
*Health‐related background variables*			
Age at diagnosis [years]	—	5.38 (3.13)	
Treatment duration [years]	—	1.34 (0.92)	
Years since cancer treatment [years]	—	4.51 (2.04)	
	*n*	*n* (%)	
Leukemia and lymphomas	0	41 (52.6)	
CNS tumors and neuroblastomas	0	17 (21.8)	
Other cancer diagnoses	0	20 (25.6)	
Surgery only	0	8 (10.3)	
Chemotherapy only	0	31 (39.7)	
Surgery and radiotherapy	0	5 (6.4)	
Surgery and chemotherapy	0	19 (24.4)	
Chemotherapy, radiotherapy and surgery	0	15 (19.2)	

Abbreviations: PCS, pediatric cancer survivors.

^a^ Age‐normed score; Mean = 100; standard deviation = 15; higher scores denote better values on the IQ scale.

**TABLE 2:** Characteristics of non‐CNS and CNS

	Non‐CNS (*n* = 61)	CNS (*n* = 17)	
	Mean (*SD*)	Mean (*SD*)	*p*
Socio‐demographic background variables			
Age [years]	10.90 (2.32)	12.42 (2.80)	0.**025**
Sex [female/male]	25/36	7/10	0.989
Height [cm]	144.61 (13.82)	146.61 (16.78)	0.583
Weight [kg]	39.46 (13.44)	45.38 (15.43)	0.125
Socioeconomic status [0–9]	6.58 (1.60)	6.68 (1.77)	0.843
Physical activity behavior [minutes/month]	638.21 (644.14)	694.76 (606.75)	0.762
Nonverbal IQ^a^	106.82 (11.90)	103.00 (10.12)	0.232
Health‐related background variables			
Age at diagnosis [years]	4.94 (3.04)	6.95 (3.05)	0.**019**
Treatment duration [years]	1.45 (0.84)	0.93 (1.09)	0.**036**
Years since cancer treatment [years]	4.50 (2.05)	4.55 (2.08)	0.939
	*n* (%)	*n* (%)	
Leukemia and lymphomas	41 (67.2)	0	
CNS tumors and neuroblastomas	0	17 (100)	
Other cancer diagnoses	20 (32.8)	0	
Surgery only	0	8 (47.1)	
Chemotherapy only	31 (50.8)	0	
Surgery and radiotherapy	1 (1.6)	4 (23.5)	
Surgery and chemotherapy	17 (27.9)	2 (11.8)	
Chemotherapy, radiotherapy and surgery	12 (19.7)	3 (17.6)	

Note

Significant group differences (*p* < .05) are indicated in bold.

Abbreviations: CNS, pediatric cancer survivors with CNS involvement; Non‐CNS, pediatric cancer survivors without CNS involvement.

^a^ Age‐normed score; Mean = 100; standard deviation = 15; higher scores denote better values on the IQ scale.

### 3.2 Comparison of PCS and TD children

On a descriptive level, TD children reached the highest average scores in all variables. With regard to inferential statistics (see Table 3 for all dependent variables; Table S2 and S3 for the full regression models), PCS showed a lower motor ability with the largest effect size in coordination (coordination: *g*_Hedges_ = 0.858; strength: *g*_Hedges_ = 0.709; aerobic fitness: *g*_Hedges_ = 0.537). A significantly lower physical self‐concept (for correlation matrix see table S4) was detected in PCS in global self‐esteem (*g*_Hedges_ = 0.366), flexibility (*g*_Hedges_ = 0.427), health (*g*_Hedges_ = 0.558), coordination (*g*_Hedges_ = 0.764), and sports competence (*g*_Hedges_ = 0.427). Notably, as in actual motor ability, largest effect sizes were found for coordination. With regard to health‐related QoL, PCS did not differ significantly from TD children (*g*_Hedges_ = 0.211).

**TABLE 3:** Regression analyses and planned Helmert contrast coding comparing motor ability, physical self‐concept and quality of life in PCS with controls and non‐CNS with CNS survivors

	Controls (*n* = 56)	Non‐CNS (*n* = 61)	CNS (*n* = 17)	Overall regression model		Controls vs. PCS		Non‐CNS vs. CNS	
	Mean (*SD*)	Mean (*SD*)	Mean (*SD*)	*p*	*f^2^*	*p*	*g*_Hedges_	*p*	*g*_Hedges_
Motor ability^a^									
Total score	101.73 (7.07)	96.25 (7.43)	91.74 (8.26)	**<0.001**	0.228	**<0.001**	−0.863	**0.** **029**	−0.591
Coordination	104.27 (9.21)	97.26 (10.06)	88.72 (12.21)	**<0.001**	0.269	**<0.001**	−0.858	**0.** **002**	−0.810
Strength	99.70 (7.83)	93.47 (9.32)	91.74 (9.34)	**<0.001**	0.148	**<0.001**	−0.709	0.103	−0.186
Physical fitness [watts]	129.23 (53.44)	101.08 (40.11)	114.75 (46.00)	**0.** **006**	0.081	**0.** **018**	−0.537	0.289	0.330
Physical self‐concept									
Global esteem	5.17 (0.66)	4.98 (0.63)	4.70 (0.87)	0.062	0.055	**0.** **023**	−0.366	0.205	−0.407
Global physical	5.17 (0.96)	5.06 (0.92)	4.68 (1.30)	0.246	0.029	0.168	−0.191	0.252	−0.376
Endurance	4.67 (0.97)	4.25 (1.20)	4.20 (1.44)	0.135	0.038	0.080	−0.377	0.892	−0.040
Strength	4.89 (0.86)	4.65 (0.97)	4.49 (1.26)	0.226	0.024	0.099	−0.279	0.578	−0.154
Coordination	5.18 (0.61)	4.67 (0.79)	4.39 (0.94)	**<0.001**	0.164	**<0.001**	−0.764	0.175	−0.340
Flexibility	5.01 (0.98)	4.61 (1.04)	4.34 (1.45)	**0.** **037**	0.053	**0.** **011**	−0.427	0.389	−0.237
Health	4.98 (1.01)	4.27 (1.17)	4.72 (0.99)	**0.** **003**	0.101	**0.** **045**	−0.558	0.221	0.397
Body fat	5.12 (1.21)	4.86 (1.44)	4.71 (1.58)	0.436	0.014	0.231	−0.213	0.703	−0.102
Sports competence	5.14 (0.82)	4.86 (0.98)	4.55 (1.20)	**0.** **048**	0.048	**0.** **017**	−0.369	0.321	−0.301
Activity	5.03 (0.98)	4.76 (1.04)	4.54 (1.42)	0.191	0.030	0.102	−0.299	0.538	−0.186
Appearance	4.40 (0.96)	4.19 (0.99)	3.62 (1.53)	**0.** **035**	0.056	**0.** **016**	−0.318	0.083	−0.506
Health‐related quality of life^b^									
Total score	53.10 (10.00)	51.80 (9.85)	47.61 (13.6)	0.205	0.029	0.124	−0.211	0.186	−0.390

Significant *p*‐values (*p* < .05) are indicated in bold.

Abbreviations: CNS, PCS with CNS involvement; Non‐CNS, PCS without CNS involvement; PCS, pediatric cancer survivors.

^a^ Age‐normed score; mean =100; standard deviation =10; higher scores denote better values in motor abilities.

^b^ Age‐normed score; mean =50; standard deviation =10; higher scores denote better values on the quality of life scale.

When looking at the likelihood for a below average motor ability (see Table S5), a larger number of PCS showed a below average motor ability (60.23%), compared to TD children (23.21%; *RR* = 1.87; 95% CI [1.39; 2.52]). In QoL ratings, a larger number of PCS had a below average QoL (15.38%), compared with TD children (5.36%; *RR* = 1.44; 95% CI [1.07, 1.95]).

### 3.3 Comparison of PCS of non‐CNS and CNS cancer

On a descriptive level, survivors after non‐CNS cancer reached highest average scores in all variables except physical fitness and health (see Table 3). With regard to inferential statistics, significant differences were found only in the motor ability domain (total score, coordination score).

When looking at the likelihood for a below average motor ability, 57.05% of survivors of non‐CNS cancer fell below the average (total score), compared to 68.24% of survivors of CNS cancer, however the *RR* is not significantly higher (*RR* = 1.58; 95% CI [0.62, 4.05]). In QoL ratings, 11.48% survivors after non‐CNS cancer had a *T*‐score ≤40 compared to 29.41% survivors of CNS cancer, however the *RR* is not significantly higher (*RR* = 2.29, 95% CI [0.99, 5.32]).

### 3.4 Potential confounders

When adding potential confounders (age, sex, socioeconomic status) to the regression models, the overall pattern of results did not change (see Table S2 and S3 for full regression models). Only in two instances, where the *p*‐value was near 0.05 before adding covariates, a statistically significant result turned nonsignificant after adding the covariates. This was the case for the comparison between TD children and PCS for the physical self‐concept facet of health and for the comparison between children after non‐CNS and CNS cancer for the motor ability total score. It therefore seems that most results are stable even when controlling for additional covariates.

### 3.5 Mediation analyses

To test the hypothesized relationship between actual motor ability (total score) and QoL, mediated by the global physical self‐concept, mediation analyses were performed (for correlation matrix including motor ability, the global physical self‐concept and QoL see Table S6). Group‐separated mediation analyses revealed a significant mediation effect in PCS (see Figure 1) and more explained variance of QoL (25.7%) compared to TD children (12.6%). In detail, in the PCS group (model 1A), results show a significant direct effect from motor ability to QoL and a significant indirect effect. In the TD group (see Figure 1, model 1B) results show a nonsignificant direct effect from motor ability to QoL and a nonsignificant indirect effect. In both groups however, a significant relationship between the physical self‐concept and QoL (path b) was found. When adding potential confounders (age, sex, socioeconomic status) to the mediation models, the pattern of results did not change (see Figure S1).

Multi‐group analyses (see Table S7) showed that when one path was constrained to equality (model 2.1–2.3), the model fit (AIC, BIC) improved only in the two models with paths a or c´ constrained (models 2.1, 2.2). Furthermore, model 3.1 (paths a and c´ constrained) overall showed the best model fit (comparative fit index [CFI] , root mean square error of approximation [RSMEA], standardized root mean square residual [SRMR]). This is also reflected by the chi‐squared difference tests, finding no significant differences in model fit of model 3.1 compared to 2.1 and 2.2. In addition, model 3.1 showed a significantly better model fit (CFI, RSMEA, SRMR, chi‐squared difference test) compared to model 4 (all three paths constrained), indicating that both groups differ only with regard to path c´.

## 4 DISCUSSION

We investigated late effects of childhood cancer and its treatment in motor ability, physical self‐concept, and QoL. First, we present evidence for impairments in motor ability in PCS, which were also reflected by a lower physical self‐concept compared to TD children. Second, we show that motor ability and physical self‐concept were linked to QoL in PCS.

The conducted mediation analyses showed that motor ability and physical self‐concept are related to QoL in PCS. This finding is in line with the conceptual model developed by Stodden et al. and the associated empirical evidence in TD children.^12^, ^13^ However, it goes beyond these previous findings by including the construct of motor ability (encompassing muscular and motor fitness), physical self‐concept and health‐related QoL in physical and nonphysical health domains. This extension highlights that also nonphysical (e.g., psychosocial) health domains may be related to actual and perceived motor ability/competence, particularly in clinical populations.

In PCS, the detected relationships of the mediation analysis were stronger and explained more variance of QoL compared to TD children. To speculate, motor deficits may prevent children from participating in physical activities during and after inpatient treatment, which in turn may have a negative impact on survivors’ QoL. Therefore, a model focusing on motor competence adopting a broader focus including the relationship with nonphysical outcomes, may be even more important and suitable for clinical populations such as PCS.

The available empirical evidence suggests that during and after the acute phase of childhood cancer, affected individuals show poorer motor ability than healthy peers.^19^, ^20^, ^60^, ^61^, ^62^ In line with these studies, we found poorer motor ability in PCS compared to controls and survivors of CNS malignancies performed poorer than those without CNS involvement. In addition, largest effect sizes were found for coordination. These results are particularly striking considering that in TD children, motor coordination was found to be associated with physical (body weight, cardiorespiratory fitness), social (social cognition, social skills) and cognitive (cognitive performance, academic achievement) development.^14^, ^15^, ^17^, ^63^, ^64^ Therefore, motor ability should be monitored closely in aftercare with a particular focus on PCS with CNS involvement.

Besides the actual motor ability, the related physical self‐concept of PCS was lower compared to TD children. Although no study has investigated the physical self‐concept in survivors, there is a previous study on acute pediatric cancer patients.^36^ The authors found significant differences only in the facets of health and flexibility. In contrast, in this study a lower physical self‐concept was detected in multiple facets including global self‐esteem, flexibility, health, coordination, and sports competence. Again, the differences between PCS and TD children were most pronounced with regard to the facet of coordination. It seems that even after the end of the acute phase, where actual and perceived motor deficits are already present,^21^, ^36^ the physical self‐concept worsens over time. A limited socialization to sports and physical activity during the illness, reduced participation rates in organized sports and physical education within 12 months after inpatient treatment (compared to TD children),^65^ may have negatively affected the physical self‐concept in PCS. This underlines the necessity to intervene early in order to preserve and promote an integer self‐concept. Therefore, and in particular for those children with a low physical self‐concept, physical activity interventions including a guided reflection on performance and improvements may be useful.^66^

The mean scores of QoL ratings were within the average range and no significant differences were found compared to TD children. This finding seems contradictory to recent systematic reviews finding lower QoL in PCS compared to TD children.^4^, ^5^ However, 29% of PCS with CNS involvement (compared to: 5% TD children; 11% non‐CNS) reached a potentially clinically significant score (≤40). Therefore, the findings of this study are in line with a previous study in Swiss PCS of comparable age,^67^ finding that PCS´ QoL was comparable to norms and QoL was lower in PCS after CNS tumors compared to non‐CNS patients. These results may indicate that although a substantial proportion shows reduced QoL, in international comparison Swiss PCS may have a better prognosis.

This study is not without limitations. First, a heterogeneous sample was included in this study. Children differed with regard to age at diagnosis, time since diagnosis, cancer type, and treatment. Even though the sample size was large enough to calculate comparisons between survivors of CNS and non‐CNS cancer, other variables such as time since diagnosis could not be considered in the analyses. Further studies are needed to investigate differential effects of cancer‐related risk factors associated with motor ability deficits. Second, only cross‐sectional data were investigated in this study. Although the mediation analysis was based on theoretical assumptions, due to the cross‐sectional design of the study, one has to be aware that the results should not be interpreted causally, and these relationships have to be tested in the future. Therefore, longitudinal studies (e.g., cross‐lagged panel designs) investigating the relationships among motor ability, self‐concept and QoL are needed. Third, although participating and nonparticipating PCS were comparable in terms of their demographic and clinical data, other interindividual differences may have influenced the decision to participate or not to participate in this time‐consuming study. Similarly, the children in the control group, who were recruited via public notice boards, might not be representative of all Swiss children. Since the current sample was not drawn randomly from a larger population but is a convenience sample (i.e., the children who agreed to participate), the results have to be interpreted cautiously with regard to the representativeness of the participants.

From the results of this study some important conclusions for future studies and clinical practice can be derived. First, monitoring and supporting the development of motor ability is important in the aftercare of PCS, in particular in patients with brain tumors. Second, effect sizes indicate that not all motor ability domains seem to be equally affected, which underlines the need for a thorough and standardized assessment of multiple motor ability domains. Third, the interrelations between motor ability, physical self‐concept and QoL indicate that physical activity is relevant to successful long‐term development. Therefore, early physical activity interventions targeting multiple motor ability domains may have a positive impact on QoL.

## 5 ETHICS APPROVAL

This study was conducted in the cantons of Bern and Zurich, Switzerland, between January 2017 and December 2018. It was granted ethics approval by the respective cantonal ethics committees (Bern: KEK‐NR. 196/15; Zurich: ZH2015‐03997) and was registered at ClinicalTrials.gov (NCT02749877).

## CONFLICT OF INTEREST

The authors do not have any conflicts of interest. The results of the study are presented clearly, honestly, and without fabrication, falsification, or inappropriate data manipulation.

## AUTHOR CONTRIBUTIONS

Conception or design of the work: JS, KL, MS, MG, MSc, RE, and VB; acquisition of data: JS, RE, VS, and VB.; data analysis: JSc and VB.; interpretation of data for the work: JSc, KL, MS, MSc, RE, and VB; draft of the work: VB. All co‐authors revised the work critically for important intellectual content and approved the final version of the manuscript. Furthermore, all co‐authors are accountable for all aspects of the work ensuring that questions related to the accuracy or integrity of any part of the work are appropriately investigated and resolved.

## Supporting information

Appendix

Figure S1

## Data Availability

Data will be made available upon reasonable request.
