# Supplementary material for: Motor ability, physical self‐concept and health‐related quality of life in pediatric cancer survivors
Source: Cancer Med. 2021 Feb 1;10(5):1860–71. doi: 10.1002/cam4.3750 (PMC7940246; doi:10.1002/cam4.3750)
Supplement: Supplementary file 1 — Appendix [file CAM4-10-1860-s002.docx]

**Table S1.** Demographic and clinical data of participating pediatric cancer survivors in comparison to those who declined to participate in the current study.

|  | **PCS**  (*n* = 78) | **Non-participating PCS**  (*n* = 161) | Test statistic | *p* |
| --- | --- | --- | --- | --- |
|  |  |  | *t*/$\chi$² |  |
|  | Mean (*SD*) | Mean (*SD*) |  |  |
| Age [years] | 11.37 (2.61) | 12.10 (2.87) | -1.90 | .058 |
| Sex [female/male] | 32/46 | 73/88 | 0.40 | .528 |
| Age at diagnosis [years] | 5.38 (3.13) | 5.85 (3.31) | 1.05 | .294 |
| Treatment duration [years] | 1.34 (0.92) | 1.29 (1.19) ^†^ | -0.30 | .768 |
| Years since cancer treatment [years] | 4.51 (2.04) | 5.03 (2.4) ^†^ | 1.62 | .107 |

*Note*. PCS = pediatric cancer survivors; ^†^ based on *n* = 151; as for 10 children the exact end of treatment is unknown. Since there is no date of participation for non-participation PCS, age was calculated using the January 1^st^, 2018 as reference date for participating and non-participating PCS.

**Table S2.** Model summaries for multiple regression models investigating differences in motor ability, physical self-concept and health-related quality of life between TD children and PCS and between non-CNS and CNS survivors.

|  |  | Model 1 | | | | |  | Model 2 | | | | | |  |
| --- | --- | --- | --- | --- | --- | --- | --- | --- | --- | --- | --- | --- | --- | --- |
|  | Variable | *R*^2^ | Corr. *R*^2^ | *SE* | *F* | *p* $\Delta$*F* |  | *R*^2^ | Corr. *R*^2^ | *SE* | $\Delta$*R*^2^ | $\Delta$*F* | *p* $\Delta$*F* |  |
| *Motor ability* | Total score | 0.19 | 0.17 | 7.39 | 14.97 | **< .001** |  | 0.23 | 0.20 | 7.27 | 0.04 | 2.44 | .069 |  |
|  | Coordination | 0.21 | 0.20 | 10.01 | 17.67 | **< .001** |  | 0.22 | 0.19 | 10.11 | 0.00 | 0.18 | .910 |  |
|  | Strength | 0.13 | 0.12 | 8.73 | 9.69 | **< .001** |  | 0.25 | 0.22 | 8.21 | 0.12 | 6.68 | **< .001** |  |
|  | Physical fitness | 0.07 | 0.06 | 46.84 | 5.28 | **.006** |  | 0.48 | 0.45 | 35.70 | 0.40 | 32.53 | **< .001** |  |
| *Physical self-concept* | Global esteem | 0.05 | 0.04 | 0.68 | 3.63 | .062 |  | 0.19 | 0.16 | 0.63 | 0.14 | 7.27 | **< .001** |  |
|  | Global physical | 0.03 | 0.01 | 0.99 | 1.92 | .246 |  | 0.11 | 0.08 | 0.96 | 0.08 | 4.08 | **.017** |  |
|  | Endurance | 0.04 | 0.02 | 1.15 | 2.53 | .135 |  | 0.14 | 0.10 | 1.10 | 0.10 | 5.04 | **.007** |  |
|  | Strength | 0.02 | 0.01 | 0.97 | 1.58 | .226 |  | 0.15 | 0.11 | 0.92 | 0.12 | 6.15 | **.002** |  |
|  | Coordination | 0.14 | 0.13 | 0.74 | 10.82 | **< .001** |  | 0.18 | 0.14 | 0.73 | 0.04 | 1.88 | .213 |  |
|  | Flexibility | 0.05 | 0.04 | 1.07 | 3.45 | **.037** |  | 0.18 | 0.15 | 1.01 | 0.13 | 6.77 | **.003** |  |
|  | Health | 0.09 | 0.08 | 1.09 | 6.63 | **.003** |  | 0.14 | 0.11 | 1.07 | 0.05 | 2.65 | .105 |  |
|  | Body fat | 0.01 | 0.00 | 1.37 | 0.91 | .436 |  | 0.11 | 0.07 | 1.32 | 0.09 | 4.50 | **.016** |  |
|  | Sports competence | 0.05 | 0.03 | 0.95 | 3.14 | **.048** |  | 0.10 | 0.06 | 0.93 | 0.05 | 2.51 | .135 |  |
|  | Activity | 0.03 | 0.01 | 1.07 | 1.96 | .191 |  | 0.10 | 0.07 | 1.04 | 0.07 | 3.55 | **.035** |  |
|  | Appearance | 0.05 | 0.04 | 1.06 | 3.68 | **.035** |  | 0.13 | 0.09 | 1.03 | 0.08 | 3.74 | **.037** |  |
| *Health-related quality of life* | Total score | 0.03 | 0.01 | 10.45 | 1.90 | .205 |  | 0.04 | 0.00 | 10.50 | 0.01 | 0.62 | .611 |  |

Note. Significant *p*-values (*p* < .05) are indicated in bold.

**Table S3.** Model parameters for multiple regression models investigating differences in motor ability, physical self-concept and health-related quality of life between TD children and PCS and between non-CNS and CNS survivors.

|  |  |  | Model 1 | | | | Model 2 | | | |
| --- | --- | --- | --- | --- | --- | --- | --- | --- | --- | --- |
|  | Variable |  | *B* | *SE B* | *t* | *p* | *B* | *SE B* | *t* | *p* |
| *Motor ability* | Total score | Constant | 96.57 | 0.77 | 125.12 | < .001 | 99.61 | 4.02 | 24.75 | < .001 |
|  |  | Healthy | 2.58 | 0.48 | 5.38 | < .001 | 2.49 | 0.48 | 5.23 | < .001 |
|  |  | CNS | 2.25 | 1.03 | 2.19 | **0.029** | 1.79 | 1.03 | 1.74 | **.083** |
|  |  | Age |  |  |  |  | -0.63 | 0.26 | -2.45 | .014 |
|  |  | FAS |  |  |  |  | 0.25 | 0.42 | 0.60 | .551 |
|  |  | Sex |  |  |  |  | 1.82 | 1.28 | 1.42 | .155 |
|  | Coordination | Constant | 96.75 | 1.06 | 91.42 | < .001 | 99.33 | 5.59 | 17.76 | < .001 |
|  |  | Healthy | 3.76 | 0.66 | 5.74 | < .001 | 3.75 | 0.67 | 5.64 | < .001 |
|  |  | CNS | 4.27 | 1.41 | 3.04 | 0.002 | 4.10 | 1.44 | 2.85 | .004 |
|  |  | Age |  |  |  |  | -0.24 | 0.36 | -0.66 | .512 |
|  |  | FAS |  |  |  |  | 0.05 | 0.59 | 0.09 | .930 |
|  |  | Sex |  |  |  |  | -0.14 | 1.79 | -0.08 | .938 |
|  | Strength | Constant | 94.80 | 0.90 | 105.55 | < .001 | 100.74 | 4.52 | 22.27 | < .001 |
|  |  | Healthy | 2.45 | 0.56 | 4.36 | < .001 | 2.27 | 0.53 | 4.26 | < .001 |
|  |  | CNS | 1.98 | 1.22 | 1.63 | 0.103 | 1.10 | 1.18 | 0.94 | .349 |
|  |  | Age |  |  |  |  | -1.20 | 0.29 | -4.12 | < .001 |
|  |  | FAS |  |  |  |  | 0.47 | 0.47 | 1.00 | .318 |
|  |  | Sex |  |  |  |  | 3.32 | 1.46 | 2.27 | .023 |
|  | Physical fitness | Constant | 115.02 | 4.77 | 24.10 | < .001 | -7.72 | 19.80 | -0.39 | .696 |
|  |  | Healthy | 7.11 | 3.00 | 2.37 | 0.018 | 8.08 | 2.31 | 3.49 | < .001 |
|  |  | CNS | -6.84 | 6.44 | -1.06 | 0.289 | 2.35 | 5.02 | 0.47 | .640 |
|  |  | Age |  |  |  |  | 12.02 | 1.28 | 9.40 | < .001 |
|  |  | FAS |  |  |  |  | 0.38 | 2.17 | 0.18 | .861 |
|  |  | Sex |  |  |  |  | -13.43 | 6.33 | -2.12 | .034 |
| *Physical self-concept* | Global esteem | Constant | 4.95 | 0.08 | 63.73 | < .001 | 6.00 | 0.37 | 16.18 | < .001 |
|  |  | Healthy | 0.11 | 0.05 | 2.31 | 0.023 | 0.10 | 0.05 | 2.21 | .030 |
|  |  | CNS | 0.14 | 0.11 | 1.28 | 0.205 | 0.07 | 0.11 | 0.60 | .551 |
|  |  | Age |  |  |  |  | -0.09 | 0.02 | -3.89 | < .001 |
|  |  | FAS |  |  |  |  | 0.03 | 0.05 | 0.59 | .568 |
|  |  | Sex |  |  |  |  | -0.11 | 0.15 | -0.74 | .467 |
|  | Global physical | Constant | 4.97 | 0.11 | 44.62 | < .001 | 6.37 | 0.55 | 11.57 | < .001 |
|  |  | Healthy | 0.10 | 0.07 | 1.39 | 0.168 | 0.10 | 0.07 | 1.42 | .159 |
|  |  | CNS | 0.19 | 0.17 | 1.16 | 0.252 | 0.12 | 0.17 | 0.71 | .482 |
|  |  | Age |  |  |  |  | -0.10 | 0.04 | -2.67 | .009 |
|  |  | FAS |  |  |  |  | 0.00 | 0.07 | -0.04 | .967 |
|  |  | Sex |  |  |  |  | -0.14 | 0.19 | -0.74 | .463 |
|  | Endurance | Constant | 4.38 | 0.16 | 27.20 | < .001 | 6.02 | 0.69 | 8.78 | < .001 |
|  |  | Healthy | 0.15 | 0.08 | 1.77 | 0.080 | 0.14 | 0.08 | 1.77 | .080 |
|  |  | CNS | 0.03 | 0.18 | 0.14 | 0.892 | -0.08 | 0.18 | -0.45 | .653 |
|  |  | Age |  |  |  |  | -0.14 | 0.04 | -3.42 | .001 |
|  |  | FAS |  |  |  |  | 0.02 | 0.07 | 0.27 | .785 |
|  |  | Sex |  |  |  |  | -0.11 | 0.21 | -0.51 | .610 |
|  | Strength | Constant | 4.68 | 0.11 | 43.01 | < .001 | 6.38 | 0.59 | 10.78 | < .001 |
|  |  | Healthy | 0.11 | 0.06 | 1.65 | 0.099 | 0.11 | 0.06 | 1.73 | .085 |
|  |  | CNS | 0.08 | 0.15 | 0.56 | 0.578 | 0.00 | 0.14 | 0.01 | .992 |
|  |  | Age |  |  |  |  | -0.11 | 0.04 | -3.04 | .003 |
|  |  | FAS |  |  |  |  | 0.00 | 0.07 | 0.00 | .999 |
|  |  | Sex |  |  |  |  | -0.33 | 0.19 | -1.79 | .077 |
|  | Coordination | Constant | 4.75 | 0.09 | 55.80 | < .001 | 5.18 | 0.43 | 11.95 | < .001 |
|  |  | Healthy | 0.22 | 0.05 | 4.03 | < .001 | 0.21 | 0.05 | 3.96 | < .001 |
|  |  | CNS | 0.14 | 0.10 | 1.36 | 0.175 | 0.10 | 0.11 | 0.94 | .348 |
|  |  | Age |  |  |  |  | -0.06 | 0.03 | -1.80 | .078 |
|  |  | FAS |  |  |  |  | 0.01 | 0.05 | 0.26 | .797 |
|  |  | Sex |  |  |  |  | 0.09 | 0.15 | 0.58 | .563 |
|  | Flexibility | Constant | 4.65 | 0.12 | 39.50 | < .001 | 5.80 | 0.63 | 9.24 | < .001 |
|  |  | Healthy | 0.18 | 0.07 | 2.55 | 0.011 | 0.16 | 0.07 | 2.43 | .015 |
|  |  | CNS | 0.14 | 0.16 | 0.86 | 0.389 | 0.03 | 0.16 | 0.17 | .866 |
|  |  | Age |  |  |  |  | -0.15 | 0.04 | -3.50 | .001 |
|  |  | FAS |  |  |  |  | 0.05 | 0.07 | 0.62 | .539 |
|  |  | Sex |  |  |  |  | 0.21 | 0.23 | 0.92 | .366 |
|  | Health | Constant | 4.66 | 0.15 | 30.61 | < .001 | 5.39 | 0.69 | 7.82 | < .001 |
|  |  | Healthy | 0.16 | 0.08 | 2.04 | **0.045** | 0.15 | 0.08 | 1.87 | **.068** |
|  |  | CNS | -0.23 | 0.18 | -1.24 | 0.221 | -0.30 | 0.19 | -1.56 | .131 |
|  |  | Age |  |  |  |  | -0.09 | 0.05 | -1.94 | .064 |
|  |  | FAS |  |  |  |  | 0.07 | 0.06 | 1.13 | .260 |
|  |  | Sex |  |  |  |  | -0.08 | 0.21 | -0.39 | .696 |
|  | Body fat | Constant | 4.90 | 0.15 | 32.59 | < .001 | 7.14 | 0.87 | 8.21 | < .001 |
|  |  | Healthy | 0.11 | 0.09 | 1.20 | 0.231 | 0.11 | 0.09 | 1.24 | .214 |
|  |  | CNS | 0.08 | 0.20 | 0.38 | 0.703 | -0.03 | 0.19 | -0.18 | .861 |
|  |  | Age |  |  |  |  | -0.13 | 0.05 | -2.44 | .018 |
|  |  | FAS |  |  |  |  | -0.05 | 0.11 | -0.50 | .624 |
|  |  | Sex |  |  |  |  | -0.26 | 0.27 | -0.97 | .337 |
|  | Sports competence | Constant | 4.85 | 0.11 | 46.06 | < .001 | 5.64 | 0.60 | 9.47 | < .001 |
|  |  | Healthy | 0.15 | 0.06 | 2.38 | 0.017 | 0.14 | 0.06 | 2.25 | .025 |
|  |  | CNS | 0.16 | 0.16 | 1.01 | 0.321 | 0.09 | 0.16 | 0.59 | .557 |
|  |  | Age |  |  |  |  | -0.08 | 0.04 | -2.04 | .049 |
|  |  | FAS |  |  |  |  | 0.03 | 0.07 | 0.41 | .682 |
|  |  | Sex |  |  |  |  | -0.01 | 0.19 | -0.04 | .972 |
|  | Activity | Constant | 4.78 | 0.13 | 37.40 | < .001 | 6.53 | 0.63 | 10.30 | < .001 |
|  |  | Healthy | 0.13 | 0.08 | 1.66 | 0.102 | 0.14 | 0.08 | 1.77 | .082 |
|  |  | CNS | 0.11 | 0.17 | 0.62 | 0.538 | 0.05 | 0.17 | 0.29 | .775 |
|  |  | Age |  |  |  |  | -0.07 | 0.04 | -1.83 | .070 |
|  |  | FAS |  |  |  |  | -0.10 | 0.07 | -1.54 | .128 |
|  |  | Sex |  |  |  |  | -0.17 | 0.21 | -0.81 | .421 |
|  | Appearance | Constant | 4.07 | 0.12 | 34.55 | < .001 | 5.41 | 0.67 | 8.13 | < .001 |
|  |  | Healthy | 0.17 | 0.07 | 2.42 | 0.016 | 0.16 | 0.07 | 2.39 | .017 |
|  |  | CNS | 0.28 | 0.16 | 1.75 | 0.083 | 0.21 | 0.16 | 1.28 | .205 |
|  |  | Age |  |  |  |  | -0.10 | 0.04 | -2.36 | .022 |
|  |  | FAS |  |  |  |  | 0.01 | 0.07 | 0.18 | .856 |
|  |  | Sex |  |  |  |  | -0.17 | 0.26 | -0.66 | .518 |
| *Health-related quality of life* | Total score | Constant | 50.84 | 1.56 | 32.65 | < .001 | 54.71 | 7.82 | 7.00 | < .001 |
|  |  | Healthy | 1.13 | 0.73 | 1.55 | 0.124 | 1.16 | 0.74 | 1.58 | .116 |
|  |  | CNS | 2.09 | 1.58 | 1.33 | 0.186 | 2.01 | 1.62 | 1.24 | .216 |
|  |  | Age |  |  |  |  | -0.11 | 0.51 | -0.21 | .838 |
|  |  | FAS |  |  |  |  | -0.32 | 0.73 | -0.43 | .669 |
|  |  | Sex |  |  |  |  | -0.38 | 2.35 | -0.16 | .873 |

*Note*. Healthy: TD children vs. PCS; CNS: non-CNS vs. CNS; Age = age in years; FAS = Family Affluence Scale; Sex: Male = 1, Female = 2. Changes in statistical significance between model 1 and model 2 are indicated in bold.

**Table S4.** Pearson correlations for the scales of the short form of the physical self-description questionnaire in PCS and TD children.

|  | 1 | 2 | 3 | 4 | 5 | 6 | 7 | 8 | 9 | 10 | 11 |
| --- | --- | --- | --- | --- | --- | --- | --- | --- | --- | --- | --- |
| 1. Global esteem | – | **.502**** | .407 | .549** | .519** | .289* | .183 | .182 | .430** | .328* | .563** |
| 1. Global physical | **.711**** | – | .501 | .608** | .576* | .300* | .216 | **.177** | .622** | .456* | .33 |
| 1. Endurance | .342* | .312 | – | **.665**** | .399** | .177 | .085 | .151 | .628** | .445** | .309 |
| 1. Strength | .574** | .473 | **.447**** | – | .520** | .239 | .228* | -.008 | **.738**** | .640** | .368** |
| 1. Coordination | .506* | .37 | .265 | .454* | – | .476** | .245 | .083 | .530* | .336** | .245 |
| 1. Flexibility | .446* | .377* | .382* | .414* | .411* | – | .208 | .111 | .224 | .212 | .217 |
| 1. Health | .340* | .284 | .093 | .109 | -.008 | .17 | – | -.153 | .042 | .068 | .019 |
| 1. Body fat | .333 | **.482**** | .146 | .118 | .204 | .198 | .131 | – | **-.016** | .032 | .229 |
| 1. Sports competence | .463** | .537* | .485** | .**537** | .307 | .378 | .024 | **.289** | – | **.588**** | **.227** |
| 1. Activity | .233 | .238 | .470** | .463** | .460* | .092 | -.162 | .14 | **.336*** | – | .202 |
| 1. Appearance | .581** | .524* | .288 | .463** | .405* | .399** | .232 | .216 | **.495**** | .135 | – |

*Note*. Correlations for typically developing children are to the left and below the diagonal. Correlations for pediatric cancer survivors are to the right and above the diagonal.

Significant correlations in the respective cohort are indicated by asterisks (* *p* < .05, ** *p* < .01).

Significant differences in correlation coefficients between the two cohorts are printed in bold (one-sided test, *p* < .05).

**Table S5.** Number of children with below average motor ability performance and potentially clinically relevant quality of life.

|  | **Controls**  (*n* = 56) | **Non-CNS**  (*n* = 61) | **CNS**  (*n* = 17) |
| --- | --- | --- | --- |
|  | *n* | *n* | *n* |
| *Motor ability* |  |  |  |
| $\leq97.5$ | 13 | 35 | 12 |
| $>$ 97.5 | 43 | 26 | 5 |
| *Health-related quality of life* |  | | |
| $<40$ | 3 | 7 | 5 |
| $\geq$ 40 | 53 | 54 | 12 |

*Note.* A higher score denotes a better value in motor ability. A motor ability score $\leq97.5$ is considered below average. A higher score denotes a better value in health-related quality of life. A motor ability score $<40$ is considered potentially clinically relevant.

**Table S6.** Pearson correlations for motor ability performance, the global physical self-concept and quality of life in PCS and TD children.

|  | 1 | 2 | 3 |
| --- | --- | --- | --- |
| 1. Motor ability performance | – | .475** | **.355**** |
| 1. Global physical self-concept | .428** | – | **.365**** |
| 1. Quality of life | **-.032** | **.005** | – |

*Note*. Correlations for typically developing children are to the left and below the diagonal. Correlations for pediatric cancer survivors are to the right and above the diagonal.

Significant correlations in the respective cohort are indicated by asterisks (* *p* < .05, ** *p* < .01).

Significant differences in correlation coefficients between the two cohorts are printed in bold (single sided test, *p* < .05).

**Table S7.** Multi-group analyses: Fit indices and test statistics (Satorra-Bentler scaled chi-squared difference test) for the different mediation models.

| **No.** | **Model** | **χ^2^** | ***p*** | **Robust** | | | **SRMR** | **AIC** | **BIC** | **Model**  **comparisons** | **Δ χ^2^** | **Δ *df*** | ***p*** |
| --- | --- | --- | --- | --- | --- | --- | --- | --- | --- | --- | --- | --- | --- |
|  |  |  |  | **χ^2^** | **CFI** | **RMSEA** |  |  |  |  |  |  |  |
| 1 | Base Model (no paths constrained) | – | – | – | – | – | – | 1052.428 | 1092.787 | – | – | – | – |
| 2.1 | Path a constrained | 2.857 | .091 | 3.674 | .956 | .178 | .070 | 1053.285 | 1090.761 | – | – | – | – |
| 2.2 | Path b constrained | 0.316 | .574 | 0.525 | 1.000 | .000 | .017 | 1050.744 | 1088.220 | – | – | – | – |
| 2.3 | Path c´ constrained | 4.180 | .041 | 6.390 | .925 | .231 | .058 | 1054.608 | 1092.084 | – | – | – | – |
| 3.1 | Paths a and b constrained | 2.948 | .229 | 3.779 | .965 | .103 | .070 | 1051.375 | 1085.969 | 3.1 vs. 2.1  3.1 vs. 2.2 | 0.116  2.750 | 1  1 | .734  .097 |
| 3.2 | Paths a and c´ constrained | 7.198 | .005 | 10.629 | .875 | .210 | .098 | 1055.625 | 1090.219 | 3.2 vs. 2.1  3.2 vs. 2.3 | 7.528  4.310 | 1  1 | .006  .038 |
| 3.3 | Paths b and c´ constrained | 4.185 | .123 | 5.675 | .942 | .167 | .059 | 1052.612 | 1087.206 | 3.3 vs. 2.2  3.3 vs. 2.3 | 4.438  0.005 | 1  1 | .035  .941 |
| 4 | Paths a, b, and c´ constrained | 7.479 | .058 | 8.836 | .895 | .158 | .109 | 1053.906 | 1085.617 | 4 vs. 3.1  4 vs. 3.2  4 vs. 3.3 | 4.628  0.237  3.094 | 1  1  1 | .031  .626  .079 |

*Note*. Path a denotes the direct of effect of motor ability performance on global physical self-concept, b the direct effect of global physical self-concept on quality of life, c’ the direct effect of motor ability performance on quality of life under statistical control of the indirect effect a×b. CFI = comparative fit index; RMSEA = root mean square error of approximation; SRMR = standardized root mean square residual; AIC = Akaike information criterion; BIC = Bayesian information criterion.
