# Supplementary material for: Motor ability, physical self‐concept and health‐related quality of life in pediatric cancer survivors
Source: Cancer Med. 2021 Feb 1;10(5):1860–71. doi: 10.1002/cam4.3750 (PMC7940246; doi:10.1002/cam4.3750)
Supplement: Supplementary file 2 — Figure S1 [file CAM4-10-1860-s001.docx]

Global physical self-concept

Motor ability

Quality of life

*a* = .240, *p* = .185 [-.038, .409]

*b* = .438, *p* = .011* [.102, .775]

*c*´ = -.202, *p* = .265 [-.557, .153]

Total effect: *c*´ + *ab* = -.121, *p* = .525 [-.493, .251]

Indirect effect: *ab* = .081, *p* = .142 [-.027, .190]

*R*^2^*:* Quality of life = .155

Global physical self-concept = .303

*Typically developing children*

**1B**

Total effect: *c*´ + *ab* = .479, *p* < .001* [.281, .676]

Indirect effect: *ab* = .157, *p* = .023* [.021, .292]

*R*^2^*:* Quality of life = .281

Global physical self-concept = .349

*c*´ = .322, *p* = .007* [.089, .554]

*b* = .323, *p* = .006* [.093, .554]

*a* = .485, *p* < .001* [.316, .654]

Global physical self-concept

Motor ability

Quality of life

*Pediatric cancer survivors*

**1A**
